# The Outcome of Early Oral Feeding Following Elective Gastrointestinal Surgery

**DOI:** 10.7759/cureus.63802

**Published:** 2024-07-04

**Authors:** GM Ishtiaq Mahmud, Md Mahamudul Hasan, Mohammad Hasnat Hakim, Nahid Hasan Rifat, Md Atiqur Rahman Bhuiyan, Tariqul Islam, Mst. Nahida Akter, Sabrina Rahman Mithila, Mir Manarat Bin Mokarram

**Affiliations:** 1 Surgery, Sir Salimullah Medical College Mitford Hospital, Dhaka, BGD; 2 Surgery, Rajshahi Medical College Hospital, Rajshahi, BGD; 3 Surgery, Dhaka Medical College Hospital, Dhaka, BGD; 4 Urology, Bangabandhu Sheikh Mujib Medical University, Dhaka, BGD; 5 Surgery, Ibn Sina Specialized Hospital, Dhaka, BGD; 6 Surgery, Kumudini Women's Medical College, Tangail, BGD; 7 Surgery, Bangabandhu Sheikh Mujib Medical University, Dhaka, BGD; 8 Surgery, Ibn Sina Medical College, Dhaka, BGD

**Keywords:** recovery, hospital stays, gastrointestinal surgery, traditional postoperative oral feeding (tof), early oral feeding (eof)

## Abstract

Background

Early oral feeding (EOF) after gastrointestinal (GI) surgery is an optimistic way to speed up recovery and shorten hospital stays, but its full effects remain unexplored.

Aim

This study aims to evaluate the outcomes of EOF in patients having elective gastrointestinal surgery.

Methods

This open-level, prospective randomized controlled trial was conducted in the Department of Surgery at Sir Salimullah Medical College Mitford Hospital, Dhaka, from March 2022 to February 2023. A total of 50 patients were enrolled and divided into two groups: early oral feeding (EOF) and traditional postoperative oral feeding (TOF), both before and after 48 hours of surgery, using a systematic random sampling technique. Informed written consent was taken from the patients. The patients were monitored on days 1, 3, 5, 7, 14, and 28 following surgeries. Postoperative complications, the duration for nasogastric tube (NGT) removal (days), the early recovery of bowel motility, and the length of the hospital stay (days) were noted.

Results

In this study, both EOF and TOF groups were found indifferent in terms of age distribution, gender ratio, or body mass index (BMI). However, significant differences emerged in postoperative outcomes. The TOF group experienced a significantly longer duration for nasogastric tube (NGT) removal and the initiation of oral feeding compared to the EOF group (P-value < 0.001). Complication rates, including nausea, vomiting, ileus, anastomotic leakage, wound infection, and pneumonia, did not exhibit statistically significant differences between the groups (P-value > 0.05). Moreover, the EOF group demonstrated an early recovery of bowel motility after surgery and shorter hospital stays compared to the TOF group (P-value < 0.05).

Conclusion

Starting oral feeding earlier does not increase complications. However, it does speed up recovery and shorten hospital stays.

## Introduction

A conventional approach in the postoperative care of patients who have gastrointestinal (GI) anastomosis surgery has been to abstain from providing oral nutrition or maintain a state of fasting until normal bowel function is regained. There is fear that providing nutrients through the gastrointestinal tract early on may increase the risk of ileus, anastomotic leakage/dehiscence, or aspiration pneumonia [1]. Postoperative fasting can result in inadequate enteral nutrition, prolonged recovery of digestive system function, and heightened vulnerability to pathogen infection, all of which can negatively impact postoperative healing and wound repair [2]. Sometimes, insufficient nutrition and decreased immune function result in severe infections, malnutrition, postoperative complications, and death, which occur as a result of anastomotic leaks after upper gastrointestinal surgery [3,4]. Nevertheless, some theories suggest that administering oral nutrition to patients during the early stages of their postoperative care could potentially reduce complications and shorten their hospitalization period [1].

Despite the proven benefits of early postoperative feeding as described in multiple studies, systematic reviews, and meta-analyses, conventional feeding schedules continue to be followed in most regions of Asia [5,6]. The Perioperative Quality Initiative (POQI), the American Society for Enhanced Recovery, and the European Society for Clinical Nutrition and Metabolism (ESPEN) recommend a departure from the traditional postoperative oral feeding (TOF) method. They advise the immediate resumption of oral nourishment following gastrointestinal surgery, which encompasses a balanced diet, oral nutritional support, and the consumption of clear liquids. This approach aims to enhance post-surgery recovery, minimize the duration of hospital stay, and decrease postoperative complications and deaths [7,8]. The Enhanced Recovery After Surgery (ERAS) protocol includes the provision of oral nourishment shortly after gastrointestinal (GI) surgery as part of the care plan [9]. Typically, within a day of the treatment, early oral feeding (EOF) begins with a liquid diet. As the patient's tolerance improves, more solid foods are introduced [10]. EOF has been demonstrated to enhance the deposition and strength of collagen in anastomotic sites, promote the healing of surgical wounds, improve patient adherence, reduce postoperative problems such as anastomotic leak and wound infection, and decrease both hospital stay and death rates [5,6,11-13]. Although there have been over 40 years of studies proving the safety of early feeding techniques in this specific group, the implementation of these findings into clinical practice is typically delayed [14].

The majority of evidence supporting improved postoperative outcomes of early oral feeding (EOF) was derived from studies undertaken in countries other than Bangladesh. Evidence is scarce regarding the outcomes of early oral feeding in the setting of elective gastrointestinal surgery in Bangladesh. This study aims to evaluate the outcomes of early oral feeding (EOF) compared to traditional postoperative oral feeding (TOF) to generate strong evidence that can support the transition to evidence-based practices in Bangladeshi hospitals. The goal is to improve overall standards of care and patient outcomes for those undergoing elective gastrointestinal surgery.

## Materials and methods

This open-level, prospective randomized controlled trial was carried out in the Department of Surgery at Sir Salimullah Medical College Mitford Hospital from March 2022 to February 2023. All the patients who were admitted to the department during the study period were the target population. Adult patients, irrespective of sex, who underwent elective gastrointestinal surgery involving anastomosis due to gastrointestinal pathology were included in the study. The patients with peritoneal cavity contamination, gastrointestinal tumors, previous gastrointestinal surgery, a history of preoperative chemotherapy or radiotherapy, and suspected organ failure were excluded. Before the study, ethical approval was obtained from the Institutional Ethics Committee of Sir Salimullah Medical College Mitford Hospital (reference number: 59.14.1100.031.18.001.22.2835).

A sample size of 50 patients (25 for EOF and TOF each) was determined using the formula for comparing two means: n = (Z_α_ + Z_β_)^2^ × (σ_1_^2^ + σ_2_^2^) / (µ_1_ - µ_2_)^2^. The calculation took into account a 95% confidence interval (CI), a power of 80%, and the mean values of 2.65 ± 0.917 and 3.4 ± 0.867 days for the resumption of oral feeding following gastrointestinal surgery in EOF and TOF, respectively [15]. A total of 50 adult patients who met the criteria for gastrointestinal surgery were chosen to participate in the trial. After providing a detailed explanation of the disease, the surgical procedure, and potential complications, informed written consent was obtained from the patients. Following the systematic random sampling technique, the patients were enrolled into two groups based on the administration of oral nutrition within 48 hours following the surgery (early oral feeding {EOF}) and after 48 hours following the surgery (traditional postoperative oral feeding {TOF}). For the patients with odd numbers, early oral feeding was the method of treatment, while for the patients with even numbers, either traditional postoperative oral feeding or late oral feeding (LOF) was the method of treatment.

Following the surgery, the group receiving early oral feeding (EOF) was provided with nutrition through a nasogastric (NG) or jejunal feeding tube and gastrostomy tube, all within 48 hours. The nasogastric tube (NGT) was removed from the patient within 24 hours of the patient waking up from anesthesia, and the nurse promptly began the patient on a clear liquid diet consisting of 30 mL/hour for 24 hours. The infusion rate was increased to 60 mL/hour within the following 12 hours. The patients were permitted to consume a diet consisting of fluids only for a period of 48 hours, after which they were gradually transitioned to a diet consisting of solid foods within the next 24 hours. If the patients were unable to accept early oral meals, the nasogastric tube was reintroduced, and the patients were handled per established standards. The patients with TOF were administered oral or enteral nutrition after 48 hours. Oral intake was withheld until the patient experienced the passage of gas or the presence of bowel sounds, at which point they were allowed to consume a clear liquid diet. The diet was subsequently advanced to solid foods as the patient's tolerance improved.

**Figure 1:**
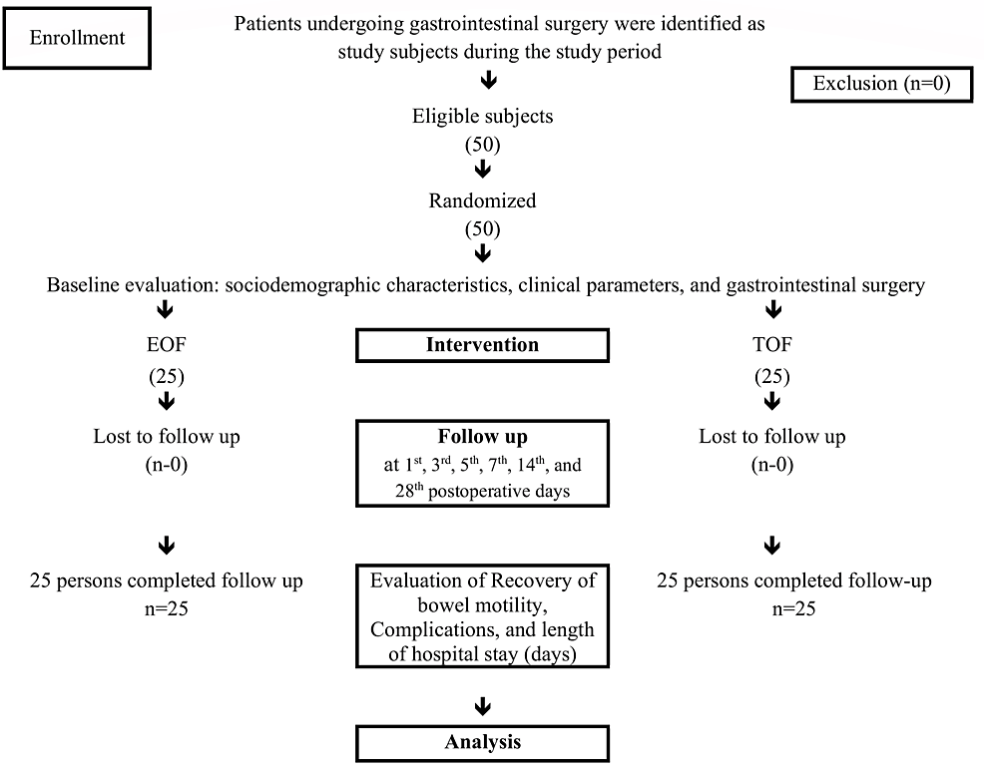
Study flow chart EOF, early oral feeding; TOF, traditional postoperative oral feeding

All patients in two groups were followed up on the first, third, fifth, seventh, 14th, and 28th postoperative days. Regular follow-up was ensured, and postoperative abdominal pain, nausea and vomiting, bowel sounds, passage of the flatus, and complications were observed (Figure 1). Flatus or stool passage was used to assess the early recovery of bowel motility. The length of hospital stays following the surgical procedure was calculated by calculating the number of days between the day of the operation and the day the patient was released from the hospital. A pretested questionnaire was utilized to collect sociodemographic, clinical, surgical, and outcome profile data.

Statistical analysis

The statistical analysis was conducted utilizing the Statistical Package for Social Science (SPSS) version 23 (IBM SPSS Statistics, Armonk, NY). To provide a summary of the continuous data, the median and range were calculated. The categorical data was presented as frequencies and percentages. The normality of the data was evaluated using the Shapiro-Wilk test. A Mann-Whitney U test was used to assess the disparity in the number of days for nasogastric tube removal, oral feeding time, and the length of hospital stays between EOF and TOF. Chi-square tests or Fisher's exact tests were conducted to ascertain the presence of a relationship between categorical variables. A P-value of <0.05 was considered acceptable as proof of statistical significance.

## Results

In our study, the majority of the patients in both groups were aged ≥30 years. The majority of the patients in both groups were male. In the EOF group, the median body mass index (BMI) was 24.7 (19.5-29.3) kg/m^2^, whereas in the TOF group, it was 25.7 (20.2-30.2) kg/m^2^. Demographically, the EOF and TOF were indifferent (P-value > 0.05) (Table 1).

**Table 1:** Distribution of the participants according to demographic characteristics (N = 50) Data was presented as frequency (percentage) and median (range) ^a^Chi-square test was done ^b^Mann-Whitney U test was done EOF, early oral feeding; TOF, traditional postoperative oral feeding

Demographic characteristics		EOF group	TOF group	P-value
		(n = 25)	(n = 25)	
Age (years)	<30	6 (24)	8 (32)	^a^0.529
	≥30	19 (76)	17 (68)	
	Median (range)	39 (21-50)	34 (21-50)	
Gender	Male	14 (56)	15 (60)	^a^0.774
	Female	11 (44)	10 (40)	
Body mass index (BMI) (kg/m^2^)	Median (range)	24.7 (19.5-29.3)	25.7 (20.2-30.2)	^b^0.396

The stomach (EOF, 40%; TOF, 36%), large intestine (EOF, 16%; TOF, 20%), and small intestine (EOF, 44%; TOF, 44%) were the major surgical sites. Gastrojejunostomy was the prevalent surgery performed in both groups. However, no significant statistical difference was seen across groups in terms of major surgical sites and surgeries performed (Table 2).

**Table 2:** Distribution of study subjects according to major surgical sites and surgeries in groups (N = 50) A chi-square test was done. Data was presented as frequency (percentage) EOF, early oral feeding; TOF, traditional postoperative oral feeding

Major surgical sites and surgeries performed		EOF group	TOF group	P-value
		(n = 25)	(n = 25)	
Site of surgery	Stomach	10 (40)	9 (36)	0.921
	Small intestine	11 (44)	11 (44)	
	Large intestine	4 (16)	5 (20)	
Surgery performed	Right hemicolectomy	2 (8)	3 (12)	0.980
	Left hemicolectomy	2 (8)	2 (8)	
	Gastrojejunostomy	10 (40)	9 (36)	
	Hepaticojejunostomy	5 (20)	6 (24)	
	Ileostomy closure	6 (24)	5 (20)	

The time for nasogastric tube removal and oral feeding was significantly longer in the TOF group compared to the EOF group (P-value < 0.001) (Table 3).

**Table 3:** Comparison of study subjects according to feeding status (N = 50) Mann-Whitney U test was done. Data was presented as median (range) EOF, early oral feeding; TOF, traditional postoperative oral feeding; NGT, nasogastric tube

Feeding status	EOF group	TOF group	P-value
	(n = 25)	(n = 25)	
NGT removal (day)	1 (1-3)	4 (2-7)	<0.001
Oral feeding time (day)	1 (1-3)	5 (3-7)	<0.001

The occurrences of nausea, vomiting, ileus, anastomotic leakage, wound infection, and pneumonia between the two groups did not show statistically significant differences (P-value > 0.05) (Table 4).

**Table 4:** Comparison of study subjects according to complications (N = 50) Fisher's exact test was done. Data was presented as frequency (percentage) EOF, early oral feeding; TOF, traditional postoperative oral feeding

Complication	EOF group	TOF group	P-value
	(n = 25)	(n = 25)	
Nausea	3 (12)	3 (12)	>0.99
Vomiting	3 (12)	3 (12)	>0.99
Ileus	2 (8)	2 (8)	>0.99
Anastomotic leakage	2 (8)	3 (12)	>0.99
Wound infection	2 (8)	7 (28)	0.138
Pneumonia	1 (4)	3 (12)	0.609

The EOF group showed statistically significant early recovery of bowel motility and shorter hospital stays compared to the TOF group (Table 5).

**Table 5:** Comparison of study subjects according to hospital outcome (N = 50) Data was presented as frequency (percentage) and median (range) ^a^Mann-Whitney U test was done ^b^Chi-square test was done EOF, early oral feeding; TOF, traditional postoperative oral feeding

Hospital outcome		EOF group	TOF group	P-value
		(n = 25)	(n = 25)	
Recovery of bowel motility (days)		3 (1-3)	4 (1-10)	^a^0.020
Length of hospital stay (days)	<8	14 (56)	5 (20)	^b^0.001
	≥8	11 (44)	20 (80)	
	Median (range)	8 (6-11)	10 (7-15)	^a^<0.001

## Discussion

Over time, traditional postoperative care for gastrointestinal (GI) surgeries involved a restriction on oral consumption until the resolution of ileus, a state marked by the passage of feces or flatus. Currently, it is recommended to resume oral food intake soon after gastrointestinal surgery including clear liquids, oral nutritional assistance, and a well-balanced diet. The main goal of early oral feeding is to enhance perioperative recovery, decrease the duration of hospitalization, and minimize the risk of postoperative complications and death [7,16]. In this study, we found EOF to enhance early recovery and reduce hospital stays in comparison to TOF. Nevertheless, there are certain similarities in the occurrence of postoperative ileus, nausea, vomiting, wound infection, and anastomotic leaking in both groups.

In our study, around 56% of the participants in the EOF group and 60% of the participants in the TOF group were male. Demographically, indifferent age and gender distribution signifies no significant impact of demographic factors on outcomes and complications between TOF and EOF. Alike demographic characteristics, no significant statistical difference was seen across groups in terms of major surgical sites and surgeries performed. In our study, the stomach, large intestine, and small intestine were the primary surgical sites, with the stomach accounting for the highest proportion, followed by the small intestine and large intestine.

In this study, it was observed that the TOF group had longer durations for nasogastric tube removal and oral feeding, in comparison to the EOF group. Shoar et al. reported that the patients in the EOF group had their nasogastric tubes removed 3.3 ± 1.6 days after surgery, while those in the late oral feeding (LOF) group had their tubes removed after 5.2 ± 2.5 days (P-value < 0.001) [17]. These findings align with our study findings.

Nevertheless, there were no notable variations observed in terms of nausea, vomiting, ileus, anastomotic leakage, wound infection, or pneumonia. The majority of randomized controlled trials found no significant differences in vomiting, nausea, or the requirement for a nasogastric tube between the groups that received early oral feeding and those that followed traditional postoperative oral feeding [18,19]. A similar observation was made with no discernible variation in oral feeding tolerance by Hosseini et al. (2010) [20]. Our study did not identify any disparities in the occurrence of postoperative pneumonia. On the other hand, a systematic review and meta-analysis conducted by Willcutts et al. (2016) [21] found that the early-fed group had a significantly lower risk of pneumonia compared to the late-fed group (odds ratio, 0.6; 95% CI, 0.41-0.89; P-value = 0.01).

The results of a systematic review and meta-analysis conducted by Deng et al. (2022) [22] and Willcutts et al. (2016) [21] demonstrated that early oral feeding, in contrast to traditional postoperative (or late) oral feeding, has the potential to reduce the duration of hospitalization and facilitate the early recovery of bowel motility without causing an increase in complications following upper gastrointestinal surgery. Like the aforementioned findings, in our study, the EOF group saw a quicker recovery of bowel movements and a shorter length of stay in the hospital in comparison to the TOF group.

Limitation

The study involved a small sample size of patients in a tertiary care hospital, which might not represent a whole-country scenario. Blinding was not utilized in this study; there may be a chance of bias, which could have influenced the outcomes. Patients with different forms and sites of gastrointestinal surgery were included in this study. However, learning the different aspects of EOF requires separate, comprehensive research in the right/left hemicolectomy, gastrojejunostomy, ileostomy closure, and hepaticojejunostomy subgroups with more patients.

## Conclusions

The early starting of oral feeding has been demonstrated to be more advantageous than the standard approach in patients who are undergoing elective gastrointestinal surgery. In contrast with traditional feeding, the early initiation of oral feeding results in shortened hospital stays following surgery and the earlier restoration of normal bowel function, with no significant increase in complications. Hence, it is practicable and safe to commence early oral feeding after elective gastrointestinal surgery.
